# Informing decisions with disparate stakeholders: cross-sector evaluation of cash transfers in Malawi

**DOI:** 10.1093/heapol/czab137

**Published:** 2021-11-18

**Authors:** Francesco Ramponi, Dominic Nkhoma, Susan Griffin

**Affiliations:** Centre for Health Economics, Alcuin A Block, University of York Heslington, York YO10 5DD, UK; Barcelona Institute for Global Health, Hospital Clínic, Universitat de Barcelona Carrer Rosselló 132, E-08036 Barcelona, Spain; Health Economics and Policy Unit, College of Medicine, Lilongwe Campus, University of Malawi, P. O. Box 3055, Lilongwe 3, Malawi; Centre for Health Economics, Alcuin A Block, University of York Heslington, York YO10 5DD, UK

**Keywords:** Economic evaluation, decision making, resource allocation, policy evaluation, research to policy, health care, education, poverty

## Abstract

The Social Cash Transfer Programme (SCTP) in Malawi is a cross-sectoral policy with impacts on health, education, nutrition, agriculture and welfare. Implementation of the SCTP requires collaboration across sectors and across national and international stakeholders. Economic evaluation can inform investment by indicating whether benefits exceed costs, but economic evaluations that provide an overall benefit–cost ratio typically assume a common agreed objective and agreed set of value judgements. In reality, the various stakeholders involved in the delivery of the SCTP may have different remits and objectives and may differ in how they value the impacts of the programme. We use the SCTP as a case study to illustrate a cross-sectoral analytical framework that accounts for these differences. The stakeholders that contribute to the SCTP include the Ministry of Gender, Ministry of Finance, Ministry of Economic Planning and Development and Global Fund. We estimate how the SCTP changes outcomes in education, health, net production and poverty, and distinguish outcomes in three groups: SCTP recipients; population in Malawi not eligible for the SCTP and population in other countries. After estimating the direct effects and opportunity costs from investing in the SCTP, we summarize the results according to different perspectives. The SCTP is estimated to provide benefits in excess of costs from the perspective of national stakeholders. From the perspective of an international donor interested in health outcomes, its health benefits do not outweigh the opportunity costs unless health improvement in SCTP recipients is valued at 18 times that of other potential spending beneficiaries or the donor values broader outcomes than health alone. This work illustrates the potential of a cross-sectoral economic evaluation to guide debate about stakeholder contributions to the SCTP, and the value judgements required to favour the SCTP above other policy options.

**Key messages**

- Using the Social Cash Transfer Programme (SCTP) in Malawi as case study, we illustrate how to conduct an economic evaluation that accommodates the perspectives of multiple heterogeneous decision makers to inform choices about investments that rely on cross-sectoral collaboration.
- This analysis brings together available research evidence on the SCTP and allows assessment of the cost-effectiveness of the programme from alternative points of view. It reflects the different goals and constraints of the various stakeholders and exposes the consequences for alternative perspectives. This information can support discussion among potential funders of the SCTP.
- We find that national stakeholders may be aligned in gaining value from investment in the SCTP, but the programme may not offer value for money for the perspective of a donor interested only in health promotion. For donors that value additional outcomes to health and/or have equity concerns that value outcomes more highly in countries with lower gross national income per capita, we show the circumstances under which the SCTP would appear to be a valuable investment.

## Introduction

### Background and objectives

Over the past two decades, increased policy attention has been given to the role that sectors other than health play in determining population health (CSDH, 2008; WHO, 2017). This has included calls for evidence and action towards strengthening collaboration across sectors, which in turn will rely on effective multi-sectoral resource allocation frameworks (Bhutta *et al.*, 2020; Forde *et al.*, 2011). Especially in low- and middle-income countries, many programmes with important health effects involve the collaboration of multiple and disparate stakeholders, including national and transnational funders, and various stakeholders across different sectors of the economy at the national and local level, including education, health and agriculture (Owusu-addo *et al.*, 2019a,b). The Social Cash Transfer Programme (SCTP) in Malawi is an example of such a policy: its impact is multidimensional, it is supported by transnational donor funding and national funds, and it is administered and overseen by multiple government departments. Collaboration across these disparate stakeholders requires that each regards the policy in question as part of their agenda and that each provides the required resource (Mcguire *et al.*, 2019; Remme *et al.*, 2017; 2014). The way that sectoral budgets are set and used in Malawi does not offer much scope for one sector to influence allocation in another. SCTP is therefore an effective case study to demonstrate how economic evaluation can recognize and inform investments that rely on cross-sectoral collaboration (Transfer Project, 2017).

Economic evaluation is used routinely in the health sector to inform investment decisions by indicating how to obtain the best value from limited resources (Drummond *et al.*, 2015). The value of a policy lies in the change in outcomes it induces. Cash transfers have been found to impact on health, health care utilization, and social determinants of health, such as poverty, education, productivity and living environment (Owusu-addo *et al.*, 2018; Pega *et al.*, 2017; Siddiqi *et al.*, 2018). Determining whether the changes in these outcomes induced by cash transfers justify the use of resources requires consideration of whether those resources could generate better outcomes if used in other ways (Sculpher *et al.*, 2017).

An economic evaluation may summarize and aggregate the impact of a policy across multiple outcomes, and all the resources employed, in order to generate an overall benefit–cost ratio or return on investment. This approach constitutes a societal perspective that requires a common agreed objective of the policy, an agreed set of relative values across each outcome and consideration of the marginal productivity of resources in different sectors in order to appropriately capture opportunity costs (Robinson *et al.*, 2019). However, if the different stakeholders involved in the delivery of the policy have different remits and objectives, they may differ in how they value the impacts of the programme (Mcdaid and Wismar, 2015; Mcdaid and Park, 2016; Forde *et al.*, 2011). The different budgetary and resource constraints between stakeholders can mean that varying how the policy is resourced has different implications in terms of value forgone from alternative activities. Under these circumstances, an economic evaluation that speaks to a notional singular decision maker may be unsatisfactory (Claxton *et al.*, 2007; 2010; Sculpher *et al.*, 2014).

In this study, we illustrate an analytical framework that lays out the various effects and makes explicit the opportunity costs to each stakeholder in order to inform about the value of a cross-sectoral policy (Walker *et al.*, 2019). This approach can indicate which stakeholders would regard a policy as value for money within their remit and budget. It can show the sensitivity of conclusions about overall value to different approaches to aggregating results across different outcomes and different population groups. Finally, it may highlight discrepancies in the marginal productivity of resources available to different stakeholders and suggest potential compensations or transfers between stakeholders. This analysis represents the first field testing of the proposed multi-sectoral approach in the context of a cash transfer programme delivered in low-income settings. The lack of multi-sectoral collaboration and coordination, however, is a problem inherent across settings. Therefore, the proposed framework for analysis could have a wide use more generally in the evaluation of other complex interventions with impacts accruing to different sectors and that involve multiple stakeholders.

### The Social Cash Transfer Programme in Malawi

Locally known as Mtukula Pakhomo, the SCTP is an unconditional cash transfer targeted to ultra-poor, labour-constrained households. The transfer amount varies by household size and the number of school-age children present in the household. The objectives of the transfer are to reduce poverty, hunger and starvation and to improve health, school enrolment and nutrition (Transfer Project, 2017). By 2018, the SCTP was operational in all the 28 districts in the country and had reached about 270 000 households and approximately 1 134 000 individuals (6% of the total population). The average amount transferred per household was MK84 000 in 2018 (approximately MK106 000 or US$145 in 2020 prices^1^) (Jha Kingra and Leach, 2019; UNICEF, 2018).

The SCTP is implemented by several branches of the Government of Malawi. These are the Ministry of Gender, Children, Disability and Social Welfare (hereafter the Ministry of Gender), the Ministry of Finance and the Ministry of Economic Planning and Development. Initial funding was largely provided by the Global Fund to Fight AIDS, Tuberculosis and Malaria. Subsequently, other donors have supported the SCTP, including the German government, the European Union, Irish Aid and the World Bank. The Government of Malawi has so far financed implementation in one district, Thyolo (Handa *et al.*, 2014; Jha Kingra and Leach, 2019).

## Methods

### Overview

We illustrate a cross-sectoral economic evaluation of the SCTP following the framework proposed by Walker *et al.* (2019). We describe the SCTP in terms of the dimensions of its value and the populations of interest to the various stakeholders involved in its implementation. Dimensions of value refer to the constituent parts of welfare that determine whether people have a good life, e.g., health and prosperity. For each dimension, we select an outcome measure. For example, to measure health we select disability-adjusted life expectancy. The overall population affected by the policy is separated into groups based on whether an individual is in receipt of the policy (directly affected), whether they are impacted by resources utilized or freed up by the policy (affected through opportunity cost) and whether they have characteristics relevant to inform equity concerns. The dimensions, outcomes and population groups determine a form of structured table called an impact matrix. Once populated with evidence, the information in the impact matrix can be collated and summarized for alternative perspectives, making explicit the value judgements required to combine impacts across dimensions and across population groups.

The main steps of evaluation are as follows: (1) define the scope of the impact matrix in terms of dimensions, outcomes and population groups; (2) populate the impact matrix and (3) aggregate impacts within and across dimensions.

1. The dimensions of the matrix are determined by identifying relevant stakeholders and decision makers, and consulting them on the appropriate dimensions and corresponding outcomes. Where stakeholders indicate that they value an outcome differently between population groups, for example due to equity concerns, the population must be subdivided by equity characteristics.
2. The matrix is populated with direct effects and opportunity costs of the policy on each outcome in each population group. To calculate opportunity costs, it is necessary to estimate the resource impact for each stakeholder, and the marginal product of the resources controlled by each stakeholder for the set of outcomes included in the matrix.
3. For stakeholders interested in a single dimension, the summary policy impact is obtained by aggregating across individuals in accordance with the relative value of the outcome in each population group. For example, if the outcome is valued equally across all individuals, the aggregation may be a simple summation. To present the summary results for perspectives that incorporate more than one dimension, the outcomes must be translated into a common unit of measurement, where the conversion reflects their relative values. It is also necessary to consider whether to aggregate first across individuals (to allow for personalized relative values) or across dimensions (where a common or societal set of relative values would be applied).

Once the last step is completed, the results of the analysis for the various perspectives can be provided to inform decision making. If direct effects net of opportunity costs are positive from all relevant perspectives, the stakeholders are aligned. However, it may happen that the net value is positive from some perspectives and negative for others. In the presence of winners and losers, consideration can be given to whether compensation or transfers could create a scenario with only winners.

### Defining the impact matrix for the SCTP

#### Relevant stakeholders

For the purpose of this illustrative study, we define a set of stakeholders based on the stated objectives of the Malawian SCTP, its sources of funding and its administration. These are the Ministry of Gender, the Ministry of Economic Planning and Development, the Ministry of Finance and donor agencies. While various donor agencies have been involved in funding the SCTP, here we present the evaluation from the perspective of the Global Fund (MOGCDSW, 2020b).

#### Dimensions of value and outcome measures

The Ministry of Gender is mandated to promote gender equality and protect the welfare of Malawians to become self-reliant and active participants of the national development agenda (MOGCDSW, 2020a). Therefore, we define health, education and poverty as dimensions of value to the Ministry. The remit of the Ministry of Economic Planning and Development is to support consumption, promote resilient livelihoods via poverty graduation pathways and develop a shock-sensitive social protection system (Government of Malawi, 2018). Therefore, we define poverty and net production as relevant dimensions of value, where net production is defined as production net of consumption. The Global Fund mobilizes and disburses resources towards ending the epidemics of AIDS, malaria and tuberculosis; we hence define health as the relevant dimension of value. The remit of the Ministry of Finance is to allocate resources across all ministries, with the objective to achieve sustainable economic growth and development (MOFEPD, 2020; Government of Malawi, 2017c). We characterize the Ministry of Finance as interested in the aggregate of all the dimensions that are relevant for the other stakeholders.

To measure the impacts in the health dimension, we select disability-adjusted life years (DALYs). These capture the impacts on health-related quality of life and length of life. DALY burdens have been estimated for undernutrition, malnutrition diarrhoea and other health events that may be influenced by the SCTP (Troeger *et al.*, 2018). To measure the impacts in the education dimension, we select the percentage of children enrolled in primary school. To measure impacts on poverty, we count the number of households below the ultra-poverty line. Finally, we measure impacts on net production using the monetary value of agricultural and non-agricultural production, consumption and savings, generated by the cash transfers.

#### Relevant population groups

The SCTP has direct effects on the recipients of the programme, and via its impact on resource use, it affects outcomes for both recipients and non-recipients. Implementing the SCTP requires funds that could have been used for other purposes. The committed financial resources could have been invested in other public policies, and the forgone benefits from these represent opportunity costs. Contrastingly, the benefits of the SCTP may include released resources, e.g., if it leads to health improvement and avoidance of health sector interventions. The benefits that could be achieved by these resources represent the opportunity gains (or negative opportunity costs). Depending on the alternative uses of the particular resource set affected, these opportunity costs or gains fall on different population groups. National (i.e. Ministry level) funds impose opportunity costs and gains on the general population in Malawi. Transnational funds impose opportunity costs and gains on individuals in other countries.

The Ministry of Gender has the remit of addressing gender equality, the well-being of vulnerable and disadvantaged groups, those with disabilities and the elderly and equitable access to child development and protection (Devex, 2020). Given the SCTP eligibility criteria, the population targeted by the programme can be considered particularly vulnerable and disadvantaged. Therefore, we assume that the eligibility criteria to receive the cash transfer describe relevant equity characteristics for the Ministry of Gender. We assume that due to equity concerns, the Ministry may value improving outcomes among SCTP recipients more than it does improving outcomes by the same amount in the general population not eligible for the SCTP. Therefore, we distinguish outcomes in each population sub-group.

In all, we identify three relevant population groups:

- SCTP recipients, experiencing the direct effects of the cash transfers and the opportunity costs or gains from the change in available resources induced by the provision of the SCTP.
- General populations in Malawi that are not eligible for the SCTP, who experience indirect effects (e.g. spill-overs) and the opportunity costs or gains from the change in resource availability to accommodate the SCTP.
- Population in other countries, impacted by opportunity costs of donor funds provided to support SCTP.

#### Structure of the SCTP impact matrix

In Table 1, we show the impact matrix that results from our defined set of stakeholders, dimensions of values and equity concerns. Dimensions and outcomes are categorized in columns, and population groups in rows. In Table 2, we illustrate the dimensions and population groups that are considered relevant for each stakeholder.

**Table 1.** Cross-sectoral impact matrix

	Dimension (Outcome)											
	Education (enrolment)			Health (DALYs averted)			Net production (MK)			Poverty (cases of ultra-poverty averted)		
Population	DE	OC	NB	DE	OC	NB	DE	OC	NB	DE	OC	NB
SCTP recipients in Malawi												
General population in Malawi not eligible for the SCTP												
Population in other countries												

DE = direct effects; OC = opportunity costs: NB = net benefits.

**Table 2.** Stakeholders’ perspectives. Dimensions of interest and relevant populations

	Dimension of interest				Relevant population	
Stakeholder	Education	Health	Net production	Poverty	Malawi	Other countries
Ministry of Gender	X	X		X	X	
Ministry of Economic Planning and Development			X	X	X	
Ministry of Finance	X	X	X	X	X	
Global Fund		X			X	X

### Populating the impact matrix for the SCTP

We use the impact matrix shown in Table 1 to evaluate the impact of the SCTP at the coverage levels observed in 2017, when cash transfer reached approximately 4% of the total population in Malawi (NSO, 2018; 2019), with over 777 000 beneficiaries in over 174 500 households across 18 districts (Transfer Project, 2017). To populate the impact matrix, we rely on a quantitative evaluation of the SCTP conducted between 2013 and 2015 as the main source of evidence of the direct effects on each outcome (Abdoulayi *et al.*, 2016; Handa *et al.*, 2014; 2015). This evaluation covered two districts (Salima and Mangochi) when the average annual transfer amount was MK26 000 in 2013 (approximately MK86 000 or US$117 in 2020 prices) (Transfer Project, 2017). We generalize the results of the pilot to the 2017 level of implementation.

Given the number of beneficiary households, we estimate a yearly cost of the transfer of approximately MK15 billion (US$20.4 million). In 2016–17, the Ministry of Gender contributed MK550 million (approximately MK944 million or US$1.3 million in 2020 prices), which included the monies transferred in one of the 18 districts, and administrative costs (UNICEF, 2017). We characterize the money for the cash transfers in the remaining 17 districts (approximately MK14 billion or US$19 million in 2020 prices) as provided by the Global Fund.

The opportunity costs on each outcome depend on the change in resource use from implementing the SCTP, and the rate at which those resources could have generated each outcome if used in alternative ways. The resources affected by the implementation of the programme include the source of the money used in the cash transfer itself, the administrative costs and the change in resource use that accompanies the change in outcomes. The rate at which each resource could have generated outcomes is its marginal product, which can be estimated empirically or inferred from the potential alternative uses of those resources. For example, where the donor could have used the supplied funds to support health-improving activities in other countries, we would seek an estimate of the rate at which the donor’s investments produce health gains. Similarly, the Ministry of Gender could have used the resources invested to cover SCTP administrative costs to fund other development-promoting activities, and so we would seek an estimate of how Ministry of Gender spend increases school enrolments across these other activities.

While estimates of the marginal productivity of health sector spend in Malawi are available (Ochalek *et al.*, 2018; Woods *et al.*, 2016), we did not find published estimates for the other sets of resources. To estimate the marginal productivities in sectors other than health care, we benchmark against other funded programmes in the sector for which we know the additional cost per additional unit of outcome gained.

Direct effects, changes in resource use and opportunity costs for each outcome are described in the following sections; detailed calculations are reported in the Supplementary Material. A summary of the inputs used for the analysis is provided in Table 3.

**Table 3.** Summary of inputs

Inputs	Value	Unit	Reference^a^
Total population			
Total population in Malawi (2017)	17 563 749	Individuals	National Statistical Office, 2018, 2019
Population aged less than 1 year	522 802	Individuals	National Statistical Office, 2019
Population aged 1–4 years	2 029 604	Individuals	NSO, 2019
Population aged 5 years	529 111	Individuals	NSO, 2019
Population aged 5–9 years	2 632 878	Individuals	NSO, 2019
Population aged 10–14 years	2 533 303	Individuals	NSO, 2019
Prevalence of diarrhoea among children aged 0–5 in the lowest wealth quintile	22.4	%	Demographic and Health Survey 2015–16 (Table 10.7)
SCTP recipients			
Number of districts where SCTP is active	18	Districts	The Transfer Project, 2017
SCTP recipients (households)	174 500	Households	The Transfer Project, 2017
SCTP recipients (individuals)	777 000	Individuals	The Transfer Project, 2017
Proportion of total population receiving SCTP	4.4	%	SCTP recipients/Total population
Unit costs			
Size of transfer (per household)	85 656	MK	The Transfer Project, 2017
	116.8	US$	
Cost per child enrolled in primary school	17 740	MK	Brossard *et al.*, 2010
	24.2	US$	
Average cost per treatment of severe diarrhoea	1506	MK	Ochalek *et al.*, 2018—Supplementary Information (average of moderate and severe)^b^
	2.1	US$	
Average cost per treatment of malnutrition	220 424	MK	Ochalek *et al.*, 2018—Supplementary Information^b^
	301	US$	
Information about the health system			
Proportion of wasting cases that receive supplementary food	7.4	%	Demographic and Health Survey 2015–16 (Table 11.10, average)
Proportion of diarrhoea cases for whom advice or treatment is sought	66.2	%	Demographic and Health Survey 2015–16 (Table 10.7, lowest wealth quintile)
Coverage rate for nutrition treatments	82	%	Ochalek *et al.*, 2018—Supplementary Information
Coverage rate for diarrhoea treatments	100	%	Ochalek *et al.*, 2018—Supplementary Information
Information about Ministry of Gender (MoG)			
Funds allocated to provision of SCTP	943.6 million 1.3 million	MK US$	UNICEF, 2017
Proportion of budget allocated to ECD	13	%	UNICEF, 2017
Proportion of budget allocated to social protection	42	%	UNICEF, 2017
Proportion of budget allocated to other activities	45	%	UNICEF, 2017
Information about the Global Fund (GF)			
Funds allocated to provision of SCTP	14.1 billion 19.3 million	MK US$	Total amount of transfer—Contribution to transfer from MoG (excluding administration costs)
Proportion of budget allocated to malaria	25	%	Micah *et al.*, 2018
Proportion of budget allocated to HIV/AIDS	50	%	Micah *et al.*, 2018
Proportion of budget allocated to tuberculosis	25	%	Micah *et al.*, 2018
Marginal productivities			
Cost to avert one additional DALY for the GF	18	US$	Shillcutt *et al.*, 2009, Laxminarayan *et al.*, 2006 (weighted average of malaria, HIV/AIDS, and tuberculosis)
Cost to avert one additional DALY for the health system	100	US$	Woods *et al.*, 2016
Cost to avert one additional DALY for the MoG	87	US$	Wilford *et al.*, 2012
Cost to generate one additional enrolment for the MoG	597	US$	Assumption informed by results of Galloway *et al.*, 2009
Cost to avert one additional ultra-poverty case for the MoG	N/A		Not available
Other			
Rate of conversion MK/US$ in 2020	733.11	MK/US$	World Bank Global Economic Monitor
Ultra-poverty line (per household)	175 470	MK	Abdoulayi *et al.*, 2016
	239.4	US$	

a Estimates are inflated to a common price year of 2020. Inflation rates and original estimates are provided in the Supplementary Material.

b Coverages and unit costs were taken from a database of cost-effectiveness evidence that was established to inform the design of the Malawian EHP. Estimate includes only costs of drugs and supply and is therefore a lower bound of the actual unit cost.

#### Direct effects and associated impacts on resource use

##### Education

In 1 year of the programme, primary school enrolment among children in SCTP households increased by 7.6 percentage points (Abdoulayi *et al.,* 2016; Dake *et al.,* 2018). If 4.4% of all children aged 6–14 reside in SCTP recipient households, this would equate to an additional 15 591 children enrolled in primary school across all households in the 18 districts.

The increase in enrolment demands additional resource use in the education sector. In Malawi, children enter primary school from age 6 and are provided with 8 years of funded primary education (World Bank, 2004). Over 90% of funding is generated within the country (Brossard *et al.*, 2010). While the Ministry for Education Science and Technology does not fund the SCTP, cash transfers do affect the demand for education resources. The average cost per student for 1 year of primary school (i.e. the annual cost per child enrolled) is MK17 740 (Brossard *et al.*, 2010). The additional 15 591 enrolees would therefore increase primary education cost by approximately MK276.6 million (US$377 000).

##### Health and nutrition

To model the impact of SCTP on health, we consider the health outcomes highlighted in the quantitative evaluation of the programme, namely: wasting, stunting and diarrhoeal episodes among children under 5. For each disease, we combine information on prevalence at the baseline, proportion of cases for which treatment is typically sought and impact of the SCTP on outcomes and treatment seeking (Abdoulayi *et al.,* 2016; NSO and ICF, 2017).

In this analysis, we use the reported point estimates that describe associations with the outcomes, regardless of their statistical significance. SCTP recipient households reported fewer cases of diarrhoea, higher likelihood of seeking treatment for diarrhoea, fewer cases of wasting and increased cases of stunting compared to non-recipient households (Abdoulayi *et al.*, 2016). From this we estimate 2317 fewer cases of diarrhoea, 2736 fewer cases of wasting and 2027 more cases of stunting among children under 5 in 1 year. A combination of fewer cases of diarrhoea but higher treatment seeking among SCTP recipients results in a net increase of 1401 cases of diarrhoea for whom advice or treatment is sought. Approximately 200 wasting cases (7%) are associated with a treatment for malnutrition (NSO & ICF, 2017). No evidence is available for the proportion of cases of stunting for which health interventions are sought.

In the Malawian health system, treatment for severe diarrhoea is included in the Essential Health Package (EHP) and provided for free (Government of Malawi, 2017b; Ochalek *et al.*, 2018; 2016). Because the treatment cost per average case of severe diarrhoea is MK1500 (US$2.00), additional costs for diarrhoea treatments incurred by the health system are on average approximately MK2.1 million. Treatment of acute malnutrition costs approximately MK220 000 (US$300). Malnutrition treatments provided for free by the EHP reach only 82% of the patients. Therefore, we assume that 82% of the 200 wasting cases that are treated are funded by the EHP; 18% are funded out-of-pocket (Ochalek *et al.*, 2018; 2016). As a result, the health system saves approximately MK36.6 million in treatments for malnutrition. We did not identify estimated treatment costs associated with stunting. In total, provision of the SCTP would save the health system approximately MK34.5 million (US$47 000) in healthcare treatments.

Diarrhoeal episodes are associated with a disability weight of 0.170 (WHO, 2018) and assumed to last for 1 week; further, they increase the risk of dying (Levine *et al.*, 2020). Stunting and wasting are associated with disability weights of 0.002 (Trenouth *et al.*, 2018) and 0.128 (WHO, 2018), respectively, and they are assumed to last for the lifetime and for 6 months, respectively. Further, they both increase mortality risk (Mcdonald *et al.*, 2013). By combining these parameters with population characteristics in Malawi (NSO, 2019), we convert variations in cases of diarrhoea into 41 845 DALYs averted, calculate 31 371 DALYs averted by reduced cases of wasting and estimate an additional 12 203 DALYs associated with increased cases of stunting. In total, 61 014 DALYs are averted (calculations in the Supplementary Material).

##### Net production

Cash transfers can directly impact recipients’ consumption and production. Moreover, beneficiary households can be a conduit through which cash is channelled into the local economy, potentially stimulating demand for retail goods, services and agricultural goods. Through such spill-overs, non-beneficiary households can also gain (Thome *et al.,* 2015; Beegle *et al.,* 2018).

In their evaluation of the SCTP in 2015, Abdoulayi *et al.* (2016) monetized all statistically significant impacts of the cash transfer on the local economy, namely, consumption, debt repayments, agricultural and non-agricultural assets, purchase of agricultural inputs and savings. By comparing the total impact on consumption and production (MK44 283) to the average transfer received by households (MK26 169), the authors estimated a multiplier effect of 1.69 in 1 year. If we assume that these estimates represent the full impact of cash transfers on net production, each MK transferred to beneficiaries generated an additional MK0.69 of worth of benefit. Further details can be found elsewhere (Abdoulayi *et al.*, 2016). We do not attempt to adjust this multiplier for scale effects of extending SCTP beyond that underlying the original evaluation. If we assume the additional benefits due to the multiplier fall equally across the population in Malawi, the funds transferred in the SCTP generate an additional gain of MK456 million for SCTP recipients and MK9.9 billion for the general population not eligible for the SCTP.

##### Poverty

Households in receipt of the SCTP were 14.9 percentage points less likely to fall below the ultra-poverty line (Abdoulayi *et al.,* 2016). Based on the average number of people in these households, this corresponds to 115 773 cases of ultra-poverty averted.

#### Opportunity costs

In Malawi, it has been estimated that each additional US$100 spent on healthcare averts 1 DALY (Ochalek *et al.*, 2018; Woods *et al.*, 2016). Therefore, the savings of MK34.5 million (US$47 000) for the health system correspond to 471 DALYs averted. If we assume that these are distributed equally across the whole population in Malawi, 21 DALYs are averted in SCTP recipients and 450 DALYs in the general population not eligible for the SCTP.

The Global Fund could have invested funds spent on the SCTP in other activities to prevent HIV/AIDS, malaria and tuberculosis in Malawi or other countries. We did not identify an estimate of the marginal health productivity (e.g. DALYs averted per dollar invested) for the Global Fund as a whole (GiveWell, 2010; 2020). Here, we benchmark the Global Fund spend against the mean incremental cost per DALY averted of activities to prevent malaria, HIV/AIDS and tuberculosis in the in sub-Saharan African region. These are one DALY averted per US$5 spent on malaria, US$1156 on HIV/AIDS, and US$5879 on tuberculosis (Laxminarayan *et al.*, 2006; Shillcutt *et al.*, 2009). We assume that the Global Fund invests 50% of its resources in activities to prevent HIV/AIDS and 25% in both malaria and tuberculosis (Micah *et al.*, 2018). Contribution to the SCTP from the Global Fund could potentially have averted approximately 1 055 000 DALYs if invested in these other activities.

Instead of investing in the SCTP, the Ministry of Gender could fund other activities to improve education and health, reduce poverty and increase net production. In 2016–17, the Ministry of Gender spent 13% of its budget on Early Childhood Development (ECD) activities, which include a broad range of programs from conception to entry into primary school; 42% on social protection and development activities^2^; 13% on activities to assist elderly and people with disabilities; 9% on probation and rehabilitation services; 4% on adult literacy activities and 19% administration costs (UNICEF, 2017; Government of Malawi, 2017a).

The impact of ECD services on health is primarily through addressing the lack of adequate nutrition and care (UNICEF, 2019). Based on previous cost-effectiveness estimates of treatments for acute malnutrition in Malawi (Wilford *et al.*, 2012; Batura *et al.*, 2015), we approximate that investments in ECD may generate health at a rate of 1 DALY per US$87. We approximate that ECD activities may promote enrolment at a rate of US$597 per additional enrolment. In lieu of direct estimates, this was extrapolated from the cost per additional day of schooling from school feeding programmes in Malawi (Galloway *et al.*, 2009). We therefore estimate a health opportunity cost of 1927 DALYs averted (85 in SCTP recipients and 1841 in the general population not eligible for SCTP) and education opportunity costs of 280 enrolments (12 in SCTP recipients, 268 in the general population not eligible for SCTP) based on the proportion of Ministry of Gender funds spent on the SCTP that may have been invested in other ECD activities.

No evidence was found about the marginal productivity of resources in terms of poverty averted, nor the cost-effectiveness of alternative social assistance programmes. Evidence suggests that cash transfers are a highly efficient means of addressing poverty that is defined in terms of income (Beegle *et al.*, 2018). Therefore, we assume that cases of ultra-poverty averted by the SCTP would match or exceed any opportunity costs. We did not find any studies to inform the rate at which activities for people with disabilities and elderly promote health. The remaining proportion of spent not translated into health and education opportunity costs is government consumption, which we capture within our estimate net production. Similarly, we assume that the additional education cost from increased enrolments represents government funded consumption, and the Ministry of Finance increases education sector funding in response to additional enrolments.

### Criteria for aggregating impacts within and across dimensions

To reflect stakeholders’ remit to focus on specific dimensions, we aggregate outcomes first within sectors by aggregating across individuals for each dimension. Then, if necessary, we aggregate across dimensions at the population level (Walker *et al.*, 2019).

We have assumed that stakeholders value outcomes equally across the population groups specified within the impact matrix, with the exception of the Ministry of Gender which values outcomes more highly if they accrue to the SCTP eligible population. To summarize net impacts in dimensions of value that lie outside of the Ministry of Gender’s purview, we simply sum outcomes across population groups. By contrast, when we aggregate the outcomes for the perspective of the Ministry of Gender, we should apply an equity weight to increase the value of outcomes falling on SCTP recipients compared to non-recipients, to symbolize the Ministry’s equity concerns.

If total net impacts are positive for all dimensions, stakeholders are aligned and the case for investment could be based on dominance criteria. In the absence of dominance, we can examine the potential for compensation mechanisms between stakeholders that might obtain positive net impacts for each dimension. Alternatively, explicit weights and relative values could be used to indicate a summary overall value of the programme. This may inform discussions among stakeholders as to whether they could support the set of values applied. These could, e.g., be based on consumption values of each outcomes; alternatively, relative weights could be elicited from stakeholders.

## Results

In Table 4 we show direct effects, opportunity costs and net benefits (obtained as direct effects net of opportunity costs) in each population group. Based on these estimates, we consider various combinations of net benefits according to the perspective of each of the stakeholders.

**Table 4.** Impact matrix

	Education			Health			Net production			Poverty		
	Enrolments			DALYs averted			MK (million)			Cases of ultra-poverty averted		
Population	DE	OC	NB	DE	OC	NB	DE	OC	NB	DE	OC	NB
SCTP recipients in Malawi	15 591	12	15 578	61 014	64^a^	60 950	15,403^c^	31^d^	15 372	115 773	N/A	115 773
General population in Malawi not eligible for the SCTP		268	−268		1391^b^	−1391	9857	670^d^	9187		N/A	N/A
Population in other countries					1 054 925	−1 054 925						
Net benefits for population of Malawi (without equity weighting)	15 310			59 558			24 559			115 773		
Total net benefits	15 310			−995 366			24 559			115 773		

DE = direct effects; OC = opportunity costs; NB = net benefits.

a Given by the sum of health system costs savings (–21 DALYs averted) and health opportunity costs of Ministry of Gender spend (85 DALYs averted).

b Given by the sum of health system costs savings (–450 DALYs averted) and health opportunity costs of Ministry of Gender spend (1841 DALYs averted).

c Given by the sum of the transfer amount (MK14 947 million) and the additional impact due to the multiplier (MK456 million).

d Given by the sum of Ministry of Education and Ministry of Gender spend.

For the population of Malawi, the SCTP is associated with additional 15 310 primary school enrolments, 59 558 DALYs averted, MK24 559 million of net production generated and 115 773 cases of ultra-poverty averted. Net impacts of the programme are positive in all dimensions considered for the perspective of the Ministry of Finance (i.e. education, health, net production and poverty), the Ministry of Gender (i.e. education, health and poverty) and the Ministry of Economic Planning and Development (i.e. net production and poverty). Any equity weight applied to increase the value of outcomes among SCTP recipients relative to outcomes among non-recipients in Malawi would simply reinforce these conclusions.

By contrast, for the perspective of the Global Fund, which includes health impacts and populations in other countries, the health opportunity costs are greater than the health benefits experienced by SCTP recipients in Malawi. In other words, funding the SCTP produces fewer DALYs than alternative Global Fund investments, resulting in a net failure to avert 995 366 DALYs for the broader population.

## Discussion

### Main findings

In this illustrative analysis, the net impacts of the SCTP are positive across dimensions considered relevant to the perspectives of national stakeholders involved in its implementation. By contrast, if the international donor funder is focussed on health, the health opportunity costs from forgoing investment in healthcare are not compensated by the health benefits of this social protection programme.

### Strengths

Our analysis brings together available research evidence on the SCTP and allows the assessment of the cost-effectiveness of the programme from alternative points of view. It breaks down direct effects and opportunity costs, distinguishing on what population groups they fall. The range of outcomes incorporated in the impact matrix may be common to cost–benefit analysis, but traditional approaches often encapsulate a notional singular decision maker with a single budget constraint and singular set of values. In the context of programmes delivered in low- and middle-income settings, the pool of decision makers to inform can be heterogeneous and may include national and regional policymakers and ministers, and transnational donor agencies. Our analysis reflects the different remits and resource constraints of the various stakeholders, makes their perspectives explicit and exposes clearly the consequences for each relevant stakeholder. It shows that the SCTP has a positive net impact regardless of the relative values that might be applied to summarize an overall impact on welfare for the national perspective of the Ministry of Finance.

### Limitations

#### Identification of relevant dimensions and outcomes

This illustrative example was undertaken without direct consultation of stakeholders involved in the SCTP to inform the selection of relevant dimensions and outcomes. We considered dimensions and outcomes based on stakeholders’ remit and agreed aims of the SCTP, but in practice dimensions should be identified via elicitation and consultation with stakeholders (MOGCDSW, 2020b). We chose, for simplicity, to present the example with the Global Fund as the only donor involved in the programme. This is consistent with how the SCTP was funded in the past, but we acknowledge that several other donor agencies such as the European Union and the World Bank have contributed to the programme over time. As their scope of action is broader than health care only, the opportunity costs associated with investments by these donors would be different from those of the Global Fund. Institutions such as the European Union and the World Bank also support interventions with impacts on sectors other than health and with effects, e.g., on education, poverty and net production. The relevant dimensions and outcomes are linked to the stakeholders involved in the programme, and their identification can be context dependent. This appraisal of cash transfers may not be directly generalizable to different settings.

#### Estimation of direct effects, resource use and opportunity costs

We acknowledge that populating the impact matrix may be a challenging task in contexts characterized by limited data availability. However, this work demonstrates that, if integrated with explicit assumptions and supported by evidence borrowed from similar contexts, it is feasible to implement this approach in low-income settings. The current transfer amount in the SCTP is higher than in our illustrative case study, and our results may not generalize to the current programme. Further, our analysis does not incorporate an assessment of uncertainty. While our aim here was to illustrate the evaluation framework, we acknowledge the importance of modelling uncertainty and investigating the robustness of results in economic evaluation to inform decision making. Finally, in our analysis, we assumed that the pilot would generalize, and that the SCTP could function at scale in the same way as was observed in the pilot. However, this may not be true as there may be more scope for inefficiencies, challenges in resource allocation and corruption within a larger, long-standing programme, compared to a pilot (Bennett *et al.*, 2018). This would make the programme more costly to achieve the same outcomes.

##### Health

There is uncertainty in how closely the health impacts we estimate reflect the overall net health impact of the SCTP. We included the impact on the cost of diarrhoea treatment, but did not reflect the health gains from additional treatment. The SCTP increased the demand for other healthcare by supporting treatment seeking in people who would otherwise have not sought care (Abdoulayi *et al.,* 2016), but information about other types of illnesses and injuries, and whether treatments were sought were not available. For the calculation of the Ministry of Gender health opportunity costs we benchmarked against healthcare activities that they have funded, and we acknowledge uncertainty in how well these estimates reflect marginal productivity. There is no published estimate of the marginal productivity of Global Fund investments. For the calculation of the health opportunity costs, we benchmarked against funded activities to combat malaria, HIV/AIDS and tuberculosis. However, this value is very uncertain and is accompanied by a sensitivity analysis when investigating the policy implications.

##### Education

The outcome measure we use to reflect education outcomes is narrow, capturing enrolment but omitting absenteeism and achievements (D’Alimonte *et al.,* 2019). To inform the opportunity costs on education from the Ministry of Gender funding, we benchmarked against school feeding programmes in Malawi aimed at promoting school attendance (Galloway *et al.,* 2009; Gelli *et al.,* 2011; Kristjansson *et al.,* 2016). While the resulting estimate suggests it would be possible to get more enrolments through alternative activities, this should be interpreted with caution given the distinction between increasing days at school and increasing enrolment. Even so, this implication is consistent with other evidence that unconditional cash transfers are less efficient in improving education outcomes in terms of years of schooling and reducing dropout rates compared to other policies (Baird *et al.,* 2011; J-PAL Policy Bulletin, 2017).

##### Poverty

We did not estimate opportunity costs from alternative activities to reduce poverty. However, it may not be unreasonable to assume that any alternative use of the same resources by the Ministry of Gender to alleviate poverty would have not averted ultra-poverty cases at a higher rate per kwacha invested (Beegle et al., 2018).

#### Modelling long-term impacts of the programme

Our economic evaluation was based on the immediate impacts of the programme, which may underestimate its value. Evidence for how the short-term outcomes we reflect in this case study translate into sustained longer-term benefits is limited, and it is plausible that a longer time period is needed for observation (Kilburn *et al.*, 2017; Dake *et al.*, 2018). For example, improved nutrition and changes in productivity might have long-term effects on health and education (Millán *et al.*, 2019; Beegle *et al.*, 2018).

In addition to such long-term effects, the SCTP may generate further indirect consequences. For example, increasing enrolment could induce households to change their expenditure patterns to accommodate school-related costs (Dillon, 2017). Moreover, as programmes are scaled up to larger regions, their impact on local economies and the feedback from the broader economy may change (Thome *et al.*, 2015).

While we did not quantify long-term impacts nor assess macroeconomic responses or account for the potential consequences of scaling up the programme, in principle the methodological framework we demonstrate could still be utilized to report the results of such an analysis.

### Policy implications

While national stakeholders may be aligned in supporting investment in the SCTP, the programme does not appear to offer value for money for the perspective of a donor interested in health outcomes. The information provided in our analysis could inform discussion about alternative funding arrangements. These could take the form of compensation across stakeholders to obtain positive net benefits in all dimensions. Our analysis indicates that the national stakeholders involved in the implementation of the SCTP could increase their funding contribution at the margin while maintaining value for money. Further, contributions could be asked from other ministries not directly involved in the SCTP, but interested in the positive outcomes, such as the Ministry of Agriculture, Ministry of Education and Ministry of Health. However, with the scale of external funding at almost 95% of the total cost, non-marginal and unrealistic changes would be required to support the SCTP from domestic funds alone.

Alternatively, the Global Fund could bring to bear equity concerns in valuing health outcomes more highly in countries with a lower gross national income per capita. While Malawi has one of the lowest gross national income per capita, health outcomes in Malawi would have to be valued about 18 times as much as those in other countries benefitting from the Global Fund in order to make the net health benefit positive. In a sensitivity analysis that investigates the impact of the uncertainty around the estimates of the health opportunity costs of the Global Fund, we observe that this value could range from 33 to 3 (calculations are reported in the Supplementary Material). The Global Fund may take a broader perspective than health. In fact, it has made significant investments in improving the health systems of low-income countries by addressing the determinants of health, besides direct activities to improve health (Samb *et al.*, 2009; Teerawattananon *et al.*, 2013). Laying bare the range of outcomes produced by the SCTP enables the Global Fund and other stakeholders to use the impact matrix to ascertain the benefit for alternative perspectives. If the non-health outcomes are of value to the Global Fund, the SCPT may be considered to provide value for money, depending on the relative weights it assigns to each outcome in the matrix. For example, if each case of poverty averted was as valuable as 8.6 DALYs averted, the SCTP may represent an important investment for the Global Fund.

## Conclusion

In this economic evaluation, we showed how to conduct a cross-sector economic evaluation aimed at informing multiple heterogeneous decision makers. The proposed framework can facilitate discussion among stakeholders and aims to inform resource allocation decisions for a set of stakeholders who work together without formal or institutional arrangements (such as donors and ministries) and who must each manage their various requirements, targets, expectations and budgetary resource constraints.

This work embraces a long-term view of strengthening and standardizing methods for the economic evaluation of interventions with impacts on multiple sectors, such as multi-sectoral programmes to improve nutrition (Levin *et al.*, 2019). The aim of this work was to illustrate how to operationalize a cross-sectoral analytical framework for the economic evaluation of a cash transfer programme conducted in low-income settings. However, because of its wider applicability, the proposed framework could contribute to standardizing methods for economic evaluation of multi-sectoral interventions across fields. With this work, by showing the strengths and potential for decision making of the proposed analysis, we aim to encourage analysts to test the feasibility and appropriateness of applying this approach with different interventions in other settings.

## Supplementary Material

czab137_Supp

## Data Availability

The data underlying this article are available in the article and in its online supplementary material.

## Endnotes

1. All Malawi kwacha estimates are inflated to a common price year of 2020 using the WorldData consumer price inflation rates for Malawi (see Supplementary Material) and converted to US dollars using the exchange rate from the World Bank Global Economic Monitor.

2. Given by the sum of the budget allocated to Social Cash Transfer (17%) and other social protection and development activities (25%).
