# Supplementary material for: Informing decisions with disparate stakeholders: cross-sector evaluation of cash transfers in Malawi
Source: Health Policy Plan. 2021 Nov 18;37(1):140–51. doi: 10.1093/heapol/czab137 (PMC8757493; doi:10.1093/heapol/czab137)
Supplement: czab137_Supp [file czab137_supp.zip › Supplementary material.docx]

# Supplementary material

## Summary of direct effects

| **Direct effects (DE)** | **Value** | **Unit** | **Reference** |
| --- | --- | --- | --- |
| **Education** |  |  |  |
| Impact on primary school enrolment | 7.6 | pp | Abdoulayi et al., 2016 (Table 11.1.1) |
| Primary school enrolments | 15,591 | Enrolments | 6-14 SCTP recipients * Impact on education |
| **Health of young children** |  |  |  |
| Impact on stunting | 2.0 | pp | Abdoulayi et al., 2016 (Table 10.1.1) |
| Impact on cases of stunting | 2,027 | Cases | 6-59 months SCTP recipients * Impact on stunting |
| Impact on wasting | -2.7 | pp | Abdoulayi et al., 2016 (Table 10.1.1) |
| Impact on cases of wasting | -2,736 | Cases | 6-59 months SCTP recipients * Impact on wasting |
| Impact on diarrhoea | -1.7 | pp | Abdoulayi et al., 2016 (Table 10.3.1) |
| Cases of diarrhoea before SCTP | 30,536 | Cases | 0-5yo SCTP recipients * Prevalence of diarrhoea |
| Cases of diarrhoea after SCTP | 28,219 | Cases | 0-5yo SCTP recipients * (Prevalence of diarrhoea + Impact on diarrhoea) |
| Impact on cases of diarrhoea | -2,317 | Cases | Cases of diarrhoea after SCTP - Cases of diarrhoea before SCTP |
| Impact on cases of diarrhoea for whom advice or treatment is sought | 1,401 | Cases | (Diarrhoea cases after SCTP * % seeking treatment * Impact on treatment seeking) - (Diarrhoea cases before SCTP * % seeking treatment) |
| DALYs | 61,014 | DALYs averted | Calculations provided in the Supplementary Material |
| **Treatment seeking** |  |  |  |
| Impact of SCTP on seeking treatment for diarrhoea | 10.4 | pp | Abdoulayi et al., 2016 (Table 10.3.1) |
| **Poverty** |  |  |  |
| Impact on ultra-poverty | -14.9 | pp | Abdoulayi et al., 2016 (table 7.2.5) |
| Cases of ultra-poverty | -115,773 | Cases | Total population * Impact on ultra-poverty |
| **Net Production** |  |  |  |
| Local economy multiplier | 1.69 | pp | Abdoulayi et al., 2016 (table 14.1) |
| Additional impact of the transfers | 10.3 bln | MK | Total amount of transfer * (multiplier - 1) |
|  | 14.1 mln | US$ |  |
| *Additional impact on SCTP recipients* | 456 mln | MK | Assuming benefits fall equally among the whole population |
| *Additional impact on general population not eligible for SCTP* | 9.9 bln | MK | Assuming benefits fall equally among the whole population |

## Summary of changes in resource use

| **Changes in resource use** | **Value** | **Unit** | **Reference** |
| --- | --- | --- | --- |
| Total cost of transfers | 14.9 bln | MK | Total households * Cash transfer amount |
|  | 20.4 mln | US$ |  |
| Global Fund spend | 14.1 bln | MK | Total amount of transfer - Contribution to transfer from MoG (excluding administration costs) |
|  | 19.3 mln | US$ |  |
| Ministry of Education spend | 276.6 mln | MK | Primary school enrolments * Cost per child enrolled in primary school |
|  | 377,255 | US$ |  |
| Ministry of Gender total spend | 943.6 mln | MK | Unicef, 2017 |
|  | 1.3 mln | US$ |  |
| *MoG spend for transfers* | 830.4 mln | MK | Total amount of transfer / 18 |
| *MoG spend for administration* | 113.2 mln | MK | Total contribution from MoG - Contribution to transfer from MoG |
| Health system total spend | -34.5 mln | MK | Sum of diarrhoea and malnutrition treatment |
|  | -47,050 | US$ |  |
| *Health system spend for diarrhoea treatment* | 2.1 mln | MK | Impact on cases of diarrhoea for whom advice or treatment is sought * Cost per treatment * Coverage rate |
| *Health system spend for malnutrition treatment* | -36.6 mln | MK | Cases of wasting * % seeking treatment * cost per treatment * coverage rate |
| Private expenditure (for health care) | -8.0 mln | MK | Cases of wasting * % seeking treatment * cost per treatment * (1-coverage rate) |
|  | -10,959 | US$ |  |

## Summary of opportunity costs

| **Opportunity costs (OC)** | **Value** | **Unit** | **Reference** |
| --- | --- | --- | --- |
| Opportunity costs Global Fund | 1,054,925 | DALYs averted | Sum of malaria, HIV/AIDS, and tuberculosis treatment |
| Opportunity costs health system | -471 | DALYs averted | Total savings for health system / marginal productivity health system |
| *OC health system falling on SCTP recipients* | -21 | DALYs averted | Assuming opportunity costs fall equally on population in Malawi |
| *OC health system falling on general population not eligible for SCTP* | -450 | DALYs averted | Assuming opportunity costs fall equally on population in Malawi |
| Health opportunity costs Ministry of Gender | 1,927 | DALYs averted | MoG spend on ECD / Marginal productivity MoG in generating health |
| *Health OC MoG falling on SCTP recipients* | 85 | DALYs averted | Assuming opportunity costs fall equally on population in Malawi |
| *Health OC MoG falling on general population not eligible for SCTP* | 1,841 | DALYs averted | Assuming opportunity costs fall equally on population in Malawi |
| Education opportunity costs Ministry of Gender | 280 | Enrolments | MoG spend on ECD / Marginal productivity MoG in generating education |
| *Education OC MoG falling on SCTP recipients* | 12 | Enrolments | Assuming opportunity costs fall equally on population in Malawi |
| *Education OC MoG falling on general population not eligible for SCTP* | 268 | Enrolments | Assuming opportunity costs fall equally on population in Malawi |
| Net production opportunity costs Ministry of Gender | 424.6 mln | MK | MoG spend on other activities |
| *Net production OC MoG falling on SCTP recipients* | 18.8 mln | MK | Assuming opportunity costs fall equally on population in Malawi |
| *Net production OC MoG falling on general population not eligible for SCTP* | 405.9 mln | MK | Assuming opportunity costs fall equally on population in Malawi |
| Poverty opportunity costs Ministry of Gender | N/A | Ultra-poverty cases averted | Not available |

## Calculation of DALYs

| **Population characteristics** | **Value** | **Reference** |
| --- | --- | --- |
| Life expectancy at birth in Malawi | 67 (f) | GBD DALYs and Hale Collaborators (2018) |
|  | 59.6 (m) |  |
| Health Adjusted Life Expectancy (HALE) at birth in Malawi | 57.8 (f) | GBD DALYs and Hale Collaborators (2018) |
|  | 52.4 (m) |  |
| Population distribution | 50.1% (f) | National Statistical Office, 2019 |
|  | 49.9% (m) |  |
| Average life expectancy at birth | 63.3 | Weighted average based on population distribution |
| Average HALE at birth | 55.1 | Weighted average based on population distribution |
| **Stunting** |  |  |
| Variation in cases of stunting | 2,027 |  |
| Disability weight | 0.002 | Trenouth et al. (2018) |
| Onset age | 2.5 | Assumed to be average of population subgroup |
| Duration of the disease (years) | 52.6 | HALE at birth - Onset age |
| *YLD* | *213* | *Cases of stunting * Disability weight * duration (years)* |
| Overall under-five mortality rate in Malawi | 49.7 | per 1,000 live birthts [Uncef data] |
| Overall under-five probability of dying in 13.5 years | 0.489 | Probability = 1- exp(-rate*t) |
| Hazard Ratio (HR) of dying due to stuting | 1.47 | McDonald et al. (2013) - Table 3 |
| Duration of the effect of SCTP on stunting | 13.5 | Assumed to be from onset age until 16 |
| Under-five mortality rate in Malawi if not suffering stunting | 33.8 | Under-five mortality rate in Malawi / HR of dying due to stunting |
| Under-five probability of dying in 13.5 years if not suffering stunting | 0.366 | Probability = 1- exp(-rate*t) |
| Under-five probability of dying in 13.5 years if suffering stunting | 0.122 | (Overall probability of dying in 13.5 years) - (Probability of dying in 13.5 years if not suffering stunting) |
| Average age of death | 6.75 | Assuming deaths occur uniformly from onset age to 16 years |
| *YLL* | *11,989* | *Cases of stunting * Prob of dying * (Average HALE - average age of death)* |
| ***DALY*** | 12,203 | YLD + YLL |
| **Wasting** |  |  |
| Variation in cases of wasting | -2,736 |  |
| Disability weight | 0.128 | WHO (2018) - Severe wasting |
| Duration of the disease (years) | 0.5 | Assumed to be 6 months |
| *YLD* | *-175* | *Cases of wasting * Disability weight * duration (years)* |
| HR of dying due to wasting | 2.3 | McDonald et al. (2013) - Table 3 |
| Under-five mortality rate in Malawi if not suffering wasting | 21.6 | Under-five mortality rate in Malawi / HR of dying due to wasting |
| Under-five probability of dying in 13.5 years if not suffering wasting | 0.253 | Probability = 1- exp(-rate*t) |
| Under-five probability of dying in 13.5 years if suffering wasting | 0.236 | (Overall probability of dying in 13.5 years) - (Probability of dying in 13.5 years if not suffering wasting) |
| Average age of death | 6.75 | Assumed to be average of population subgroup |
| *YLL* | *-31,196* | *Cases of wasting * Prob of dying * (Average HALE - average age of death)* |
| ***DALY*** | ***-31,371*** | ***YLD + YLL*** |
| **Diarrhoeal episodes** |  |  |
| Variation in cases of diarrhoea | -2,317 |  |
| Disability weight | 0.170 | WHO (2018) - Average of mild, moderate and severe |
| Duration of the disease (years) | 0.019 | Assumed to be 7 days |
| *YLD* | *-8* | *Cases of diarrhoea * Disability weight * duration (years)* |
| HR of dying due to diarrhoea | 5.47 | Levine et al. (2020) - Average of mild, moderate and severe |
| Under-five mortality rate in Malawi if not suffering diarrhoea | 9.1 | Under-five mortality rate in Malawi / HR of dying due to diarrhoea |
| Under-five probability of dying in 13.5 years if not suffering diarrhoea | 0.115 | Probability = 1- exp(-rate*t) |
| Under-five probability of dying in 13.5 years if suffering diarrhoea | 0.373 | (Overall probability of dying in 13.5 years) - (Probability of dying in 13.5 years if not suffering diarrhoea) |
| Average age of death | 6.75 | Assumed to be average of population subgroup |
| *YLL* | *-41,838* | *Cases of diarrhoea * Prob of dying * (Average HALE - average age of death)* |
| ***DALY*** | ***-41,845*** | ***YLD + YLL*** |
| **Total DALYs** | **-61,014** | **DALYs Stunting + DALYs Wasting + DALYs Diarrhoea** |

## Inflation and exchange rates

| **Year** | **Malawi inflation rate*** | **MK/US$ exchange rate**** |
| --- | --- | --- |
| 2000 | 29.58% | 59.38 |
| 2001 | 22.70% | 72.28 |
| 2002 | 14.74% | 76.21 |
| 2003 | 9.58% | 96.37 |
| 2004 | 11.43% | 108.39 |
| 2005 | 15.41% | 118.06 |
| 2006 | 13.97% | 136.03 |
| 2007 | 7.95% | 140.00 |
| 2008 | 8.71% | 140.52 |
| 2009 | 8.42% | 141.14 |
| 2010 | 7.41% | 150.71 |
| 2011 | 7.62% | 156.91 |
| 2012 | 21.27% | 246.02 |
| 2013 | 27.28% | 364.46 |
| 2014 | 23.79% | 421.83 |
| 2015 | 21.87% | 498.59 |
| 2016 | 21.71% | 713.28 |
| 2017 | 11.54% | 725.35 |
| 2018 | 12.42% | 725.84 |
| 2019 | 12.42% | 735.50 |
| 2020 |  | 733.11 |
|  |  |  |
| References: | |  |
| [* https://www.worlddata.info/africa/malawi/inflation-rates.php](https://www.worlddata.info/africa/malawi/inflation-rates.php) | | |
| ** World Bank Global Economic Monitor (GEM) | | |

## Inflation adjustment

| **Inputs** | **Original value** | **Unit** | **Year** | **Reference** | **Value in 2020** |
| --- | --- | --- | --- | --- | --- |
| Size of transfer (per household) | 26,000 | MK | 2013 | The Transfer Project, 2017 | 85,656 |
| Size of transfer (per household) | 84,000 | MK | 2018 | Jha Kingra & Leach, 2019 | 106,161 |
| Cost per child enrolled in primary school | 3,019 | MK | 2007 | Brossard, Coury & Mambo, 2010 | 17,740 |
| Cost per average case of treatment of severe diarrhoea | 326 | MK | 2010 | Ochalek et al., 2018 (average of moderate and severe) | 1,506 |
| Cost per average case of treatment of malnutrition | 47,729 | MK | 2010 | Ochalek et al., 2018 | 220,425 |
| Ultra-poverty line | 53,262 | MK | 2013 | Abdoulayi et al., 2016 | 175,470 |
|  |  |  |  |  |  |
| **Change in resource use** | **Original value** | **Unit** | **Year** | **Reference** | **Value in 2020** |
| MoG total spend | 550,000,000 | MK | 2016 | Unicef, 2017 | 943,640,895 |
|  |  |  |  |  |  |
| **Costs per outcome** | **Original value** | **Unit** | **Year** | **Reference** | **Value in 2020** |
| Cost per DALY averted for malaria prevention (min) | 2 | US$ | 2008 | Shillcutt et al., 2009, Laxminarayan et al., 2006 | 2 |
| Cost per DALY averted for malaria prevention (max) | 29 | US$ | 2008 | Shillcutt et al., 2009, Laxminarayan et al., 2006 | 30 |
| Average for malaria |  |  |  | 1/average(1/min cost + 1/max cost) | 5 |
| Cost per DALY averted for HIV/AIDS treatment (min) | 804 | US$ | 2008 | Shillcutt et al., 2009, Laxminarayan et al., 2006 | 838 |
| Cost per DALY averted for HIV/AIDS treatment (max) | 1,784 | US$ | 2008 | Shillcutt et al., 2009, Laxminarayan et al., 2006 | 1,861 |
| Average for HIV/AIDS |  |  |  | 1/average(1/min cost + 1/max cost) | 1,156 |
| Cost per DALY averted for tuberculosis treatment (min) | 4,930 | US$ | 2008 | Shillcutt et al., 2009, Laxminarayan et al., 2006 | 5,143 |
| Cost per DALY averted for tuberculosis treatment (max) | 6,574 | US$ | 2008 | Shillcutt et al., 2009, Laxminarayan et al., 2006 | 6,859 |
| Average for tuberculosis |  |  |  | 1/average(1/min cost + 1/max cost) | 5,879 |
| Cost per DALY averted for the MoG (min) | 42 | US$ | 2007 | Wilford et al. 2012 | 47 |
| Cost per DALY averted for the MoG (max) | 493 | US$ | 2007 | Wilford et al. 2012 | 553 |
| Average cost per DALY averted for the MoG |  |  |  | 1/average(1/min cost + 1/max cost) | 87 |
| Cost per additional enrolment for the MoG | 480 | US$ | 2005 | Galloway et al., 2009 (assuming 200 school days a year and US$2.40 per additional day attendance) | 597 |
| Cost to avert one additional DALY for the health system | 61 | US$ | 2013 | Woods et al., 2016 | 100 |

## Sensitivity analysis

| **Parameter** | **Average** | **Min** | **Max** | **Unit** | **Reference** |
| --- | --- | --- | --- | --- | --- |
| Cost to avert one additional DALY for the GF | 18 | 10 | 115 | US$ | Shillcutt et al., 2009, Laxminarayan et al., 2006 (weighted average of malaria, HIV/AIDS, and tuberculosis) |
| Opportunity costs Global Fund | 1,054,925 | 1,942,930 | 166,919 | DALYs averted | Sum of malaria, HIV/AIDS, and tuberculosis treatment |

Scenario 1: minimum cost to avert one additional DALY for the Global Fund

| **Dimensions 🡪** | **Education** | | | **Health** | | | **Net production** | | | **Poverty** | | |
| --- | --- | --- | --- | --- | --- | --- | --- | --- | --- | --- | --- | --- |
| **Outcomes 🡪** | **Enrolments** | | | **DALYs averted** | | | **MK (million)** | | | **Cases of ultra-poverty averted** | | |
| **Population** | **DE** | **OC** | **NB** | **DE** | **OC** | **NB** | **DE** | **OC** | **NB** | **DE** | **OC** | **NB** |
| SCTP recipients in Malawi | 15,591 | 12 | 15,578 | 61,014 | 64 | 60,950 | 15,403 | 31 | 15,372 | 115,773 | N/A | 115,773 |
| General population in Malawi not eligible for the SCTP |  | 268 | -268 |  | 1,391 | -1,391 | 9,857 | 670 | 9,187 |  | N/A | N/A |
| Population in other countries |  |  |  |  | 1,942,930 | -1,942,930 |  |  |  |  |  |  |
| **Net Benefits for population of Malawi (without equity weighting)** | **15,310** | | | **59,558** | | | **24,559** | | | **115,773** | | |
| **Total Net Benefits** | **15,310** | | | **-1,883,371** | | | **24,559** | | | **115,773** | | |

Health outcomes in Malawi would have to be valued about (1,942,930 / 59,558 =) 33 times as much as those in other countries benefitting from the Global Fund in order to make the net health benefit positive

Scenario 2: maximum cost to avert one additional DALY for the Global Fund

| **Dimensions 🡪** | **Education** | | | **Health** | | | **Net production** | | | **Poverty** | | |
| --- | --- | --- | --- | --- | --- | --- | --- | --- | --- | --- | --- | --- |
| **Outcomes 🡪** | **Enrolments** | | | **DALYs averted** | | | **MK (million)** | | | **Cases of ultra-poverty averted** | | |
| **Population** | **DE** | **OC** | **NB** | **DE** | **OC** | **NB** | **DE** | **OC** | **NB** | **DE** | **OC** | **NB** |
| SCTP recipients in Malawi | 15,591 | 12 | 15,578 | 61,014 | 64 | 60,950 | 15,403 | 31 | 15,372 | 115,773 | N/A | 115,773 |
| General population in Malawi not eligible for the SCTP |  | 268 | -268 |  | 1,391 | -1,391 | 9,857 | 670 | 9,187 |  | N/A | N/A |
| Population in other countries |  |  |  |  | 166,919 | -166,919 |  |  |  |  |  |  |
| **Net Benefits for population of Malawi (without equity weighting)** | **15,310** | | | **59,558** | | | **24,559** | | | **115,773** | | |
| **Total Net Benefits** | **15,310** | | | **-107,361** | | | **24,559** | | | **115,773** | | |

Health outcomes in Malawi would have to be valued about (166,919 / 59,558 =) 3 times as much as those in other countries benefitting from the Global Fund in order to make the net health benefit positive
